# Skin Biter: Dermatodaxia Revisited

**DOI:** 10.7759/cureus.22289

**Published:** 2022-02-16

**Authors:** Philip R Cohen

**Affiliations:** 1 Dermatology, University of California, Davis Medical Center, Sacramento, USA

**Keywords:** wolf, repetitive, dermatophagia, dermatodaxia, skin, chew, body, biter, bite, behavior

## Abstract

Dermatodaxia describes humans who bite their skin. Previously used designations, which are less appropriate, have included chewing pads, wolf-biter, and dermatophagia. Dermatodaxia is a body-focused repetitive behavior and is classified in the category of obsessive-compulsive and related disorders. People who bite their skin may concurrently have other related disorders such as dermatillomania (also referred to as skin picking) affecting their cutaneous integument, trichotillomania (also referred to as hair-pulling) affecting their hair, and/or onychophagia (also referred to as nail-biting) affecting their nails. A man with multiple medical conditions presented for follow-up evaluation of a recently treated superficial skin infection of his abdomen. Cutaneous examination not only showed complete resolution of an abdominal abscess but also dermatodaxia involving his dorsal left index finger; the skin biting site appeared as an asymptomatic lichenified nodule with overlying scaly hyperkeratosis. Additional inquiry confirmed that for several decades he would repeatedly bite this finger. He was aware - and even demonstrated - that his skin biting caused the lesion. He also declined any interventions to alter his behavior. Similar to the patient in this report, dermatodaxia typically presents as an asymptomatic, unilateral, solitary, lichenified, callous-like, thick nodule; however, bilateral involvement or multiple biting sites or both may occur. Lesions typically occur on the forearm, hands, and fingers; on the latter site, they may or may not involve the knuckles. Physical modalities, behavior modifications, and/or pharmacologic agents may be used in the management of dermatodaxia; however, many individuals with dermatodaxia - similar to the reported man - are not only aware that the cutaneous lesion is caused by their skin biting but also do not want to entertain the possibility of initiating any intervention that might change or stop their skin biting.

## Introduction

Skin biting is a condition in which the affected individual repeatedly bites themselves. The affected individual was originally described as a wolf-biter. However, this terminology was initially revised to designate the disorder as dermatophagia and subsequently changed to the more appropriate term dermatodaxia [1-6].

Dermatodaxia is categorized as an obsessive-compulsive and related disorder. It describes people who habitually bite their skin but do not consume the skin they have bitten. Dermatodaxia can be an isolated body-focused repetitive behavior or occur with other self-induced dermatoses [7-11].

A patient with dermatodaxia is described as a 41-year-old man who repetitively bites his left index finger. He was aware of his skin biting and did not want to consider any intervention. Other reports of patients with dermatodaxia are summarized and the possible interventions for this condition are reviewed.

## Case presentation

A 41-year-old man presented for follow-up of a resolved abscess on his abdomen. Four weeks earlier he was seen in the emergency room for an infection on the left lower abdomen that had spontaneously ruptured and was draining; he was concurrently treated for 10 days with cephalexin 500 mg four times daily and trimethoprim-sulfamethoxazole double-strength twice daily. The abscess healed completely.

His past medical history was significant for depression, diabetes (type 2), hypercholesterolemia, hypertension, and sleep apnea. His current medication included aspirin (enteric-coated), glimepiride, linsinopril, metformin, metoprolol succinate (extended-release), paroxetine, and simvastatin. He had multiple prior surgeries at the following sites: left arm, nose (rhinoplasty), right shoulder (rotator cuff repair), and skull.

**Figure 1:**
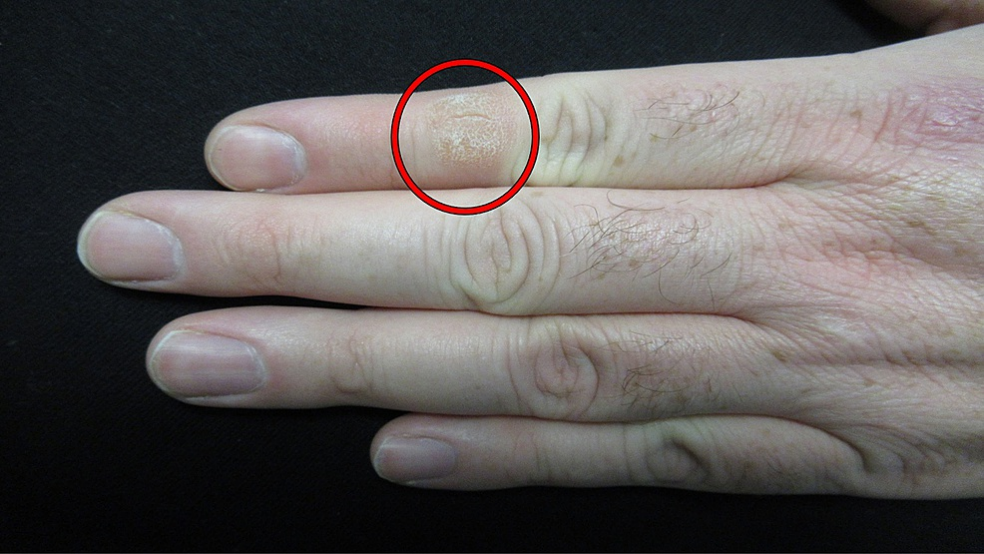
Dermatodaxia presenting as a digital nodule A distal view of the fingers on the dorsal left hand of a 41-year-old man. The left index finger shows an asymptomatic 12 x 12 millimeter scaly hyperkeratotic nodule (red oval) between the knuckles of his proximal and distal interphalangeal joints.

**Figure 2:**
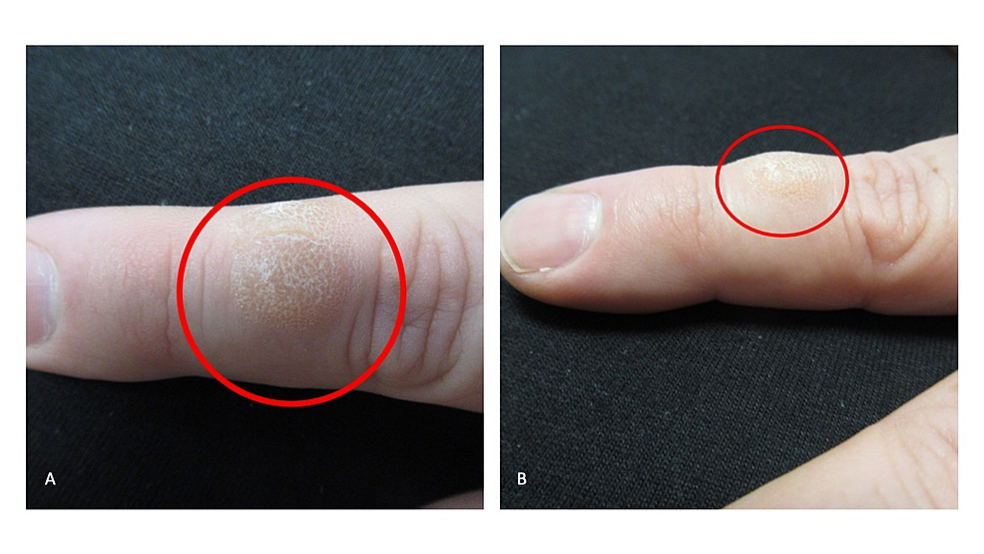
Skin biting resulting in a nodule on the dorsal left second digit Superior (A) and lateral (B) views of the left index finger show a lichenified nodule with overlying superficial scaly hyperkeratosis (red oval) between the proximal and distal knuckles.

**Figure 3:**
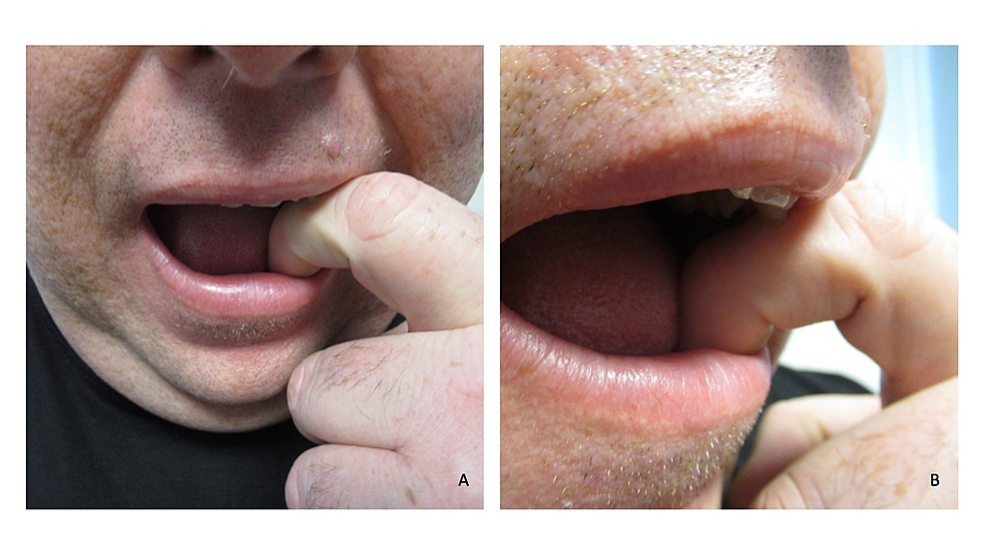
The patient demonstrated the technique he would use to bite the skin of his left index finger Frontal (A) and right-sided (B) views show the placement of the patient’s second digit in his mouth when he would bite his left index finger. Dermatodaxia is an obsessive-compulsive disorder and skin biting is classified as a body-focused repetitive behavior.

Cutaneous examination showed complete healing of the prior abscess on his left lower abdomen with hyperpigmentation at the site. His left hand showed a 12 x 12 millimeter, lichenified nodule with overlying scaly hyperkeratosis on the dorsal index finger (second digit) between the knuckles of his proximal and distal interphalangeal joints (Figures 1, 2A, 2B). Additional history revealed that for several decades he has had a habit of repetitively biting this finger (Figures 3A, 3B).

Correlation of the history and clinical presentation established the diagnosis of dermatodaxia (skin biting). Occasionally, the bite site on his finger would get irritated. However, he did not want to consider any behavioral, physical, or systemic intervention.

## Discussion

Drs. Marion B. Sulzberger and Sadie H. Zaidens are credited for calling attention to the existence of compulsive movement-associated cutaneous lesions in humans. In 1948, they summarized their observations on psychogenic factors in dermatologic disorders and those described in the literature. In the section on psychogenic dermatoses and symptom complexes, they commented not only that “lesions resulting from compulsive movements can occur in any site…and are due to biting, chewing, [and] sucking…” but also that they “…have seen skin lesions from compulsive movements or tics on almost every part of the integument--from toes to the crown” [12].

Animals have also been observed to self-inflict injury. For example, monkeys are tail-chewers and may only have five or six inches of tail remaining after they have chewed away their distal tail. In addition, wolves - if annoyed, frustrated or trapped - may bite themselves [4,5].

Similar behavior to that noted in animals had also been observed in humans who would bite or chew their skin; indeed, the patient was referred to as a wolf-biter. The origin of this descriptive term was provided by Dr. Frederick DeForest Weidman, a dermatologist and dermatopathologist, during the discussion of a paper presented by Dr. Albert R. McFarland on skin diseases influenced by mechanical trauma at the 72nd Annual Meeting of the American Dermatological Association in Colorado Springs, Colorado, on April 25, 1952. Dr. Weidman not only credited Dr. Butterworth with introducing the distinctive nomenclature but also commented on the specific location of the lesions observed by Dr. Butterworth; however, according to statements subsequently made by Dr. Butterworth, Dr. Weidman’s designation of the bite site location was incorrect [4,13].

Dr. Weidman specifically stated that “As to wolf-biters, this designation was given by Dr. Thomas Butterworth, who is a consultant to a state institution for the feeble-minded. There he found that many of the inmates chew their knuckles. However, there are many people who lead monotonous lives and do not live in institutions. I recall that at one of our Philadelphia Dermatological Society meetings Dr. Butterworth suggested that diagnosis for a patient who was not an inmate of an institution…” [4].

In 1976, Meigel and Plewig described the condition as chewing pads. Biting, chewing, or sucking resulted in a pad-like thickening localized to the affected areas: the knuckles. The researchers considered the condition to be a variant of knuckle pads [14,15].

Subsequently, in 1997, Scott and Scott introduced the term dermatophagia to replace the earlier designation of wolf-biter. Dermatophagia describes the condition of an individual with a compulsion or habit, either conscious or subconscious, that results in that person biting their own skin. The researchers considered this condition analogous to other self-mutilating disorders such as hair pulling or nail biting [5].

Several years later in 2005, Mitropoulos and Norton evaluated the term dermatophagia. Indeed, dermatophagia implied not only biting but also consumption of the skin. Although there are reptiles and amphibians that display dermatophagia by eating their shed skin, most humans only bite the skin [6].

The investigators then searched ancient and modern Greek dictionaries; they discovered that “dermato” refers to “of the skin” and “daxia” refers to “the act of biting.” Hence, Mitropoulos and Norton suggested using the nomenclature dermatodaxia when only biting of the skin is occurring [6]. Therefore, this paper shall use the more appropriate terminology dermatodaxia when referring to all patients who were published with this condition - whether they were described as having dermatodaxia or a less accurate nomenclature such as dermatophagia or chewing pads or wolf biter.

A new classification of obsessive-compulsive and related disorders was not only adopted by the American Psychiatric Association publication of Diagnostic and Statistical Manual of Mental Disorders, Fifth Edition (DSM5) on May 18, 2013 but also incorporated into the Mental and Behavioral Disorders chapter of the Eleventh Revision of the World Health Organization’s International Classification of Diseases and Related Health Problems (ICD-11) which was initiated on January 1, 2022. Specifically, there is now a section that is referred to as body-focused repetitive behaviors. This section includes not only dermatotillomania (also known as skin picking) and trichotillomania (also known as hair-pulling), but also dermatodaxia [8,9].

Dermatodaxia occurs not only as a solitary obsessive-compulsive condition but also concurrently with other body-focused repetitive behaviors. Houghton et al. studied a group of 4,435 undergraduate university students, from June 2014 to May 2017, for the following body-focused repetitive behaviors: cheek biting, hair pulling, nail-biting, skin biting, skin picking, and teeth grinding. They discovered that 72% (3,185 students) had a body-focused repetitive behavior within the current or past month [10]. 

However, the body-focused repetitive behavior in Houghton et al.’s study was subclinical in 60% (2,641 students) of the participants. It only included one body-focused repetitive behavior (47%, 1,496 students) or concurrently involved either two (35%, 1,101 students), three (15%, 483 students), four (3%, 90 students), five (0.4%, 14 students), or six (0.02%, one student) body-focused repetitive behaviors. Skin biting was either subclinical in 2.4% (108 students) of the participants or clinical in 0.5% (21 students) of the participants [10].

Another study, from May 2002 to February 2018, was conducted at the Wisconsin Psychocutaneous Clinic in Middleton, Wisconsin, to evaluate referring and final diagnoses. A total of 808 individuals were evaluated. Several of the individuals had more than one final diagnosis [11].

Skin picking disorder was the most frequent final diagnosis in 52% (417 individuals) of the referrals. Some of the skin pickers had two or three concurrent final diagnoses. For example, either trichotillomania (8.9%, 37 individuals) or onychotillomania (2.4%, 10 individuals) were simultaneously present; and there was one person (0.2%) who had not only picked at the skin but also at both the hair and nails [11].

Investigators noticed that the sites most frequently bitten were the upper extremities - particularly, the dermatodaxia sites were the extensor forearm, dorsal hands, and fingers. However, some individuals demonstrated biting off their feet and ankles. Also, some of the patients bite their fingertips or nail folds [1-3,7].

Several reports have described a predilection for patients biting their dorsal fingers [5,14-17]. However, skin biting has also been observed on the knuckle of the thumb [13]. Indeed, the location of skin biting on the finger was variable - either involving the skin between the knuckles (similar to the patient in this report) or overlying the knuckles [5,14-16].

Many of the patients with dermatodaxia only had a unilateral and solitary site of involvement. However, multiple unilateral sites had also been described. In addition, patients with bilateral involvement had also been observed [1,3,5].

Repetitive biting of an individual skin site eventually results in a lichenified, callous-like thick nodule. In addition to hypertrophy of the skin, hyperpigmentation and hypertrichosis have been observed at the bite sites of some of the patients. More severe biting has resulted in ulcers and infection [1-3,5,16,17].

Investigation on dermatodaxia has originated from several nations: Germany, Iraq, Saudi Arabia, Switzerland, and the United States of America [1-3,5,7,10,11,14-17]. However, there is a paucity of published reports of skin biting in the world literature. Therefore, the details from some of these papers are summarized.

Some of the earliest reports of skin biting were described by Dr. Thomas Butterworth and several co-investigators. Dr. Butterworth was the dermatology consultant at the Pennhurst State School and Hospital (which was originally opened on November 23, 1908, as the Eastern Pennsylvania State Institution for the Feeble-Minded and Epileptic and subsequently closed on December 9, 1987) in Spring City, Pennsylvania; this institution treated and housed mentally and physically disabled patients. Therefore, Dr. Butterworth’s observations were biased by the patient population he evaluated and treated [1-3].

Drs. Butterworth and Bower identified patients who lived at the Pennhurst State School and Hospital who frequently bit themselves or demonstrated violent chewing of their skin. They noted that skin biting occurred in approximately 2% of the institutionalized mentally challenged individuals. It affected both sexes equally and occurred at any age - but more frequently in patients ranging from 10 years to 29 years [1].

Dr. Butterworth’s papers included numerous images of not only self-inflicted bites but also hypertrophic lesions and hyperpigmentation - with or without hypertrichosis - at the bite sites [1-3]. Several years after his papers had been published, Dr. Butterworth provided further insights regarding skin biting during the discussion of a paper on knuckle pads that was presented by Drs. J Richard Allision, Jr. and J Richard Allision, Sr. on February 26, 1965, at the 85th Annual Meeting of the American Dermatological Association. He commented that knuckle pads “…are not seen among the mentally retarded who bite their fingers, hands, and forearms” and that “this group usually does not chew the knuckles” [13].

During his comments, Dr. Butterworth also had shared some additional observations regarding patients with dermatodaxia. He mentioned that “…salesmen occasionally drive along biting a knuckle…when they stop for traffic lights” and that “the proximal interphalangeal joint of the index finger on the left hand is chewed most commonly.” He also described a high-strung executive with a callus over the interphalangeal joint of his thumb who would chew the site at board meetings and when concentrating; the habit persisted, even after persuasion to stop [13].

A localized variant of dermatodaxia, referred to as chewing pads, was restricted to the knuckles and morphologically mimicked knuckle pads. They were described by Meigel and Plewig in three young men who each had a tic-like habit that consisted of sucking and/or biting on the dorsal proximal joints of the thumb and fingers. The chewing habit resulted in a thickening of the affected sites [14].

The youngest boy was 14 years old with a one-year duration of asymptomatic swollen proximal interphalangeal joints with overlying thickening of the ring finger on both hands. After an extensive negative systemic evaluation, he admitted - during additional inquiry - to chewing the back of the fourth digits while concentrating or doing schoolwork. The cause of the lesions was explained to the patient; at a follow-up visit three months later, he was noted to be chewing his fingers less often and the chewing pads were smaller [14].

The second boy was 17 years old; his mother had noticed, for about the prior six months, that the proximal interphalangeal joints on the second, third, fourth, and fifth digits on his left hand were swollen and thickened. After an exhaustive negative systemic workup, additional history was obtained from the patient; the boy stated that he would chew on these fingers during exciting situations and while doing schoolwork. His physicians referred him for an in-depth psychological examination and the evaluators recommended psychotherapy; however, the boy declined this management and - after a single follow-up visit four weeks later - did not return [14].

The third patient was a 20-year-old man who was being evaluated and treated for serology-positive syphilis-associated phimosis; during his initial examination, thickening of the skin over the proximal interphalangeal joins of not only the thumb but also the second, fifth - and a lesser degree, third - digits was noted. The patient commented that he constantly chewed on these fingers, especially the index finger which was most severely affected. His latent syphilis infection was treated with 30 days of tardocillin and no intervention was provided for the chewing pads [14].

Several years later, Wollina commented about chewing pads. He mentioned that chewing pads could still be observed in obsessive-compulsive computer and video game users and that they resulted from the mechanical factors caused by the repetitive biting, chewing, or sucking of the affected skin site. Wollina also discussed that the surface of the affected skin thickens and becomes rough as a sequalae to the repetitive behavior; however, he had noted that the lesions may completely resolve if the causative behavior was modified or discontinued [15].

The 45-year-old woman reported by Scott and Scott readily admitted to her skin-biting habit and did not request any treatment for her condition. Her dorsal right hand had several callous-like, lichenified, discoid lesions not only over the proximal interphalangeal joints of all fingers but also over the knuckles of the metacarpal-phalangeal joints of her index and middle fingers. Similar to the man in this paper, she willingly demonstrated her dermatodaxia technique which involved using her upper and lowers frontal teeth to firmly hold the skin she was biting [5].

Two Middle Eastern boys were also described whose dermatodaxia resulted in lesions that simulated calloses. One was 15 years old, who had otherwise good behavior and satisfactory school performance, with thick skin-colored discoidal plaques on the knuckle prominences of both hands for several months that were initially diagnosed as calluses; when there was no improvement after three months of keratolytics, the boy’s father provided an additional history that his son had an intermittent habit of biting his knuckles. After the cause of his condition was explained to him, the boy consciously attempted to stop biting his knuckles and had almost given up the dermatodaxia during the next few months [16].

The second patient was a 16-year-old boy with chronic, bilateral, dark brown, slowly increasing in size, asymptomatic hyperkeratotic 1.5 x 1.5-centimeter plaques, with a rough surface and thick adherent scale, of at least a one-year duration on the dorsal proximal index fingers between the metacarpal-phalangeal joint and the proximal interphalangeal joint. One year prior to the onset of the skin lesions, the boy’s father had been killed by militants; within a short period of time, his behavior changed and he began to repeatedly bite and chew both of his second digits at the affected sites. He was diagnosed as having dermatodaxia-induced calluses; management included topical agents (a keratolytic and a moisturizing ointment) and psychiatry referral [17].

Moritz et al. reported a 50-year-old man who had not only dermatodaxia but also pathological skin picking and onychophagia since early childhood. At age 40 years, his nail-biting was considerably reduced after he was treated with a method referred to as decoupling; essentially, he would perform exercises using a physical movement to generate a conscious irritant just prior to completing the undesired behavior. However, he would still bite and pick at the skin on his fingertips and nail folds occasionally even causing bleeding; therefore, a revised decoupling protocol (involving exercises that incorporated both an imaginal and a behavioral sequence) was initiated that resulted in eliminating both his skin biting and picking [7].

Several approaches for the management of dermatodaxia have been introduced. These include physical modalities, behavioral modifications, and pharmacologic interventions [18-20]. However, similar to the man described in this report, many patients are not only aware of the problem and its etiology, but also strongly opposed to any intervention [5,10].

Physical modalities are those that incorporate an agent (such as a band-aid, paper tape, or a glove) to physically cover the affected skin site. Hence, by protecting the affected area, the individual is not able to bite that site. For example, band-aids or paper tape could be applied to the knuckles of the fingers involved by dermatodaxia to prevent the patient from biting the skin at these locations [18].

Protective gloves - referred to as protecting little and adolescent youth (PLAY) Hands - can be created (either using an orthosis for either the affected finger or for all fingers of the entire glove) using thermoplastics. The PLAY Hands is an intervention, using protective handwear, that has been considered for children with cerebral palsy to prevent self-mutilating behavior. However, these protective gloves could also be useful for patients with dermatodaxia localized to the hands and fingers [18].

Several behavior modifications have been used in patients with obsessive-compulsive and related disorders including body-focused repetitive behaviors such as dermatodaxia. They have had variable rates of success. Examples of these therapies include decoupling, habit reversal training, and hypnosis [7,19,20].

Pharmacotherapy is also a treatment option for patients with debilitating body-focused repetitive behaviors. Some of the medications that have been used include buspirone, clomipramine, lithium, risperidone, and selective serotonin reuptake inhibitors (such as fluoxetine, paroxetine, and sertraline) [19,20]. However, the treatment of depression with paroxetine for the man in this report did not result in any improvement of his dermatodaxia.

The man in this report had several features similar to those individuals previously described with dermatodaxia. His condition was unilateral and solitary, it was usually asymptomatic, he was aware the lesions were created by his skin biting, and he did not want to consider any potential therapeutic interventions. He was taking several medications which were unlikely to be causing his skin biting; however, whether his depression (that was being treated with paroxetine) was associated with either persistence or exacerbation of his dermatodaxia is unknown.

## Conclusions

Dermatodaxia refers to skin biting in humans. Individuals who bite their skin may concurrently have other body-focused repetitive behavior-related conditions affecting their hair, nails, and/or skin. Similar to the man in this report, dermatodaxia is usually asymptomatic, unilateral, and affects a solitary site. Individual lesions typically appear as lichenified, callous-like, thick nodules. The forearm, hands, and fingers are the most commonly dermatodaxia sites; the knuckles may or may not be involved. The management of dermatodaxia may include physical modalities, behavior modifications, and/or pharmacologic agents. Similar to the reported man, many of the individuals with dermatodaxia are not only aware of their skin biting lesion and its etiology but also do not desire any intervention to modify or eliminate the biting of their skin.
